# The construction and validation of an instrument for the assessment of graduates of undergraduate nursing courses**^1^**

**DOI:** 10.1590/1518-8345.0834.2710

**Published:** 2016-06-14

**Authors:** Maria Aparecida Vieira, Conceição Vieira da Silva Ohara, Edvane Birelo Lopes de Domenico

**Affiliations:** 2 PhD, Professor, Departamento de Enfermagem, Universidade Estadual de Montes Claros, Montes Claros, MG, Brazil.; 3 PhD, Associate Professor, Escola Paulista de Enfermagem, Universidade Federal de São Paulo, São Paulo, SP, Brazil.; 4 PhD, Adjunct Professor, Departamento de Enfermagem Clínica e Cirúrgica, Escola Paulista de Enfermagem, Universidade Federal de São Paulo, São Paulo, SP, Brazil.

**Keywords:** Validation Studies, Nurses, Nursing Assessment, Education, Nursing

## Abstract

**Objective:**

to construct an instrument for the assessment of graduates of undergraduate nursing courses and to validate this instrument through the consensus of specialists.

**Method:**

methodological study. In order to elaborate the instrument, documental analysis and a literature review were undertaken. Validation took place through use of the Delphi Conference, between September 2012 and September 2013, in which 36 specialists from Brazilian Nursing participated. In order to analyze reliability, the Cronbach alpha coefficient, the item/total correlation, and the Pearson correlation coefficient were calculated.

**Results:**

the instrument was constructed with the participation of specialist nurses representing all regions of Brazil, with experience in lecturing and research. The first Delphi round led to changes in the first instrument, which was restructured and submitted to another round, with a response rate of 94.44%. In the second round, the instrument was validated with a Cronbach alpha of 0.75.

**Conclusion:**

the final instrument possessed three dimensions related to the characterization of the graduate, insertion in the job market, and evaluation of the professional training process. This instrument may be used across the territory of Brazil as it is based on the curricular guidelines and contributes to the process of regulation of the quality of the undergraduate courses in Nursing.

## Introduction

Education is a social right and the duty of the state, according to the Brazilian Constitution. This principle is the basis of the educational institutions' social responsibility. The Higher Education Institutions (HEI) must provide society with evidence that they are meeting their responsibilities, in relation to the academic-scientific, professional, ethical and political training of citizens, to the production of knowledge, and to the promotion of the advance of science and culture^1^. 

In this context, it becomes necessary to assess courses, a complex aspect in the panorama of university education, given that the quality of these courses, including those of training in Nursing, involves multiple dimensions which must have interconnections and show the concerns of the profession and of the community in which this is inserted, taking into account the concrete reality and the future to be constructed^2^ ^-^ ^3^. In the process of assessing the adequacy of higher education training, the graduate is an important source of information which must be an element in composing other initiatives of institutional evaluation^4^.

The practice of the assessment of graduates in Brazilian national territory is, as yet, incipient, considering the accelerated and uncontrolled growth of courses and the offering of places on undergraduate Nursing courses, without the appropriate monitoring of their quality and the different sociodemographic contexts found in Brazil, in spite of the existence of relevant studies with graduates of Nursing courses which raised important evidence for the elaboration of other investigations regarding Education in Nursing. It is necessary to extend these in both public and private teaching institutions, so as to investigate sources of difficulty and what is done well, the coping mechanisms, and how the training contributes during this process; the specific characteristics of the process of training in Nursing in the different regions of Brazil so as to provide support for improvements which could improve the pedagogical practices in this trajectory, and the professional development of the Nurse^2^ ^,^ ^5^ ^-^ ^7^.

Investigating the graduates' professional trajectory is a way of analyzing, understanding and reflecting on questions relating to the teaching of Nursing and to the characteristics inherent to the institution's formative potential, society's requirements, the final product of the pedagogical work and the absorption of these professionals into the labor market. The search for excellence requires continuous assessment, so as to undertake the adjustments, constructions and reformulations in the teaching-learning process^5^ ^-^ ^6^. It is based on the knowledge of the real influence of higher education in Nursing, in the insertion of the graduate in the job market, of the strengths and weaknesses of this training in the professional career, that one can qualitatively assess the effectiveness of the undergraduate courses in the training of critical and reflexive subjects able to deal with the social and ethical challenges posed by the exercising of their profession in the world today^5^. 

Governmental studies and publications described in one review, published in the period 2001 - 2011 showed great diversity relating to data collection instruments in the search to assess the graduate in relation to her professional training^8^. It was ascertained that, in these publications, these instruments emphasize specific aspects to the detriment of others, which lacked indicators for recognizing and identifying the contribution of the course in the ambit of the purpose of HEIs and of society; and there was valorization, sometimes excessive, of quantitative data, which provided few possibilities for the graduates to aggregate their perceptions regarding the composition and relevance of the set of elements evaluated, so as to produce a holistic and integrated view of the course. It was also observed that, besides the lack of consensus of the evaluative items and content contained in these data collection instruments, these agreed little with the premises contained in the National Curricular Guidelines for the area of Nursing (DCN/ENF) which, since 2001, must define curricular pedagogical proposals in Brazil^8^ ^-^ ^9^. 

These findings revealed gaps in relation to the assessment of the Nursing courses, based on the view of graduates, and, above all, in consonance with the premises contained in the DNC/ENF. In this regard, the following research question was formulated: what content or assessment criteria must be found in an instrument for the evaluation of graduates of Undergraduate Courses in Nursing? 

This study's objectives were: to construct a data collection instrument for the evaluation of graduates from Undergraduate Courses in Nursing and to validate this instrument through the consensus of a group of specialists. 

## Method

### Study design

Methodological study, made up of two parts: construction of a data collection instrument for the evaluation of graduates of Nursing, and the validation of this instrument through the use of the Delphi technique, which seeks consensus of opinions of a group of specialists through validations articulated in phases, cycles and rounds^10^. In this study, a rate of 70% was adopted as a minimum consensus level, in the final stage of the Delphi technique^11^ ^-^ ^12^.

The validation of the instrument was undertaken by a group of specialists, made up of Brazilian nurses who were invited to participate in this study. The criteria for selecting the specialists were as follows: to be a nurse, to have professional experience in educational processes in the area of Nursing, to be linked to teaching, research or care institutions or to Nursing unions, in Brazil, to have - at the least - the title of Doctor, and to accept to be a member of the group of specialists through signing the Terms of Free and Informed Consent (TCLE).

To this end, at an initial point, 53 academics from the area of education in Nursing in Brazil were selected, these being sent invitation letters, containing information on the objectives and description of this study, and their rights as participants. Of these, 36 nurses responded positively regarding the intention of contributing to this study and returned the signed TCLE, accepting to participate in the Delphi Conference. 

### Procedure for data collection 

Data collection began in September 2012, when the first Delphi round began, following the approval of the Research Ethics Committee of the Federal University of São Paulo (UNIFESP) - Opinion N.1997/10, and continued until September 2013, when the second Delphi round was concluded. 

The instrument of the first round was developed based on a broad review of the literature in order to detect and obtain information regarding the knowledge produced until that point on this issue^8^. This instrument contained three dimensions: the first, to investigate the personal and professional profile of the graduate - *Characterization of the Graduate* - had 10 closed questions. The second (with 8 closed questions) and the third dimension (with 5 open questions) were termed *Evaluation of the Professional Training Process*, aiming to assess the knowledge required for exercising the profession, in conformity with what is stipulated by the DCN/ENF^9^. Even taking into account the actual significance of the guidelines in the professional training in Nursing, one must be aware of the consistencies and inconsistencies described in the scientific literature regarding the DCN/ENF^13^ ^-^ ^14^. 

In this regard, the present investigation was based on these guidelines, respecting them as guiding the professional training in undergraduate nursing courses on Brazilian territory. It is understood that, for the process of assessment of graduates, the DCN/ENF, at the time of writing, constitutes a major document and this study's results could help researchers and educators, also, to recognize their strengths and weaknesses. 

In order to respond to the closed questions of the questionnaire, referent to the *Evaluation of the Professional Training Process*, a scale of the Likert type was used, consisting of a set of items presented as statements or opinions, to which the participants' reactions are requested, that is, each statement is presented and the subject is requested to show her reaction, choosing one of the points or categories of the scale^15^. The varying degrees of agreement of the Likert scale, in this study, were as follows: 1 - agree completely; 2 - agree; 3 - neither agree nor disagree; 4 - disagree and 5 - disagree completely. 

The above-mentioned instrument was sent to the 36 specialists, using the LimeSurvey software, accompanied by the Delphi Panel Participants' User ID and instructions on how to proceed to the evaluation for its validation. In order for the specialists to be able to judge each question presented in this first questionnaire, spaces were inserted alongside each one of them; furthermore, places were set aside for inserting any comments and suggestions as might be thought necessary. 

After receiving the questionnaires of the first round, these were analyzed statistically in order to determine reliability and validity. Based on this analysis, a second questionnaire was developed, reflecting the information provided by the specialists in the first Delphi round for associating the principal arguments with the different tendencies of the responses. Also sent was a summary of the Delphi Group's previous stage, in order to reduce the "semantic noise", to avoid the respondents deviating from the issue's central points^16^ and the specific instructions for this second questionnaire. 

### Data analysis 

In order to analyze the data from the questionnaires of the first and second rounds of the Delphi technique, the percentage, for comparing the agreement among specialists, the Cronbach alpha coefficient, for measuring the internal consistency between the items of the questionnaire, and the Pearson correlation coefficient, for analyzing the relation between each one of the three dimensions found in the questionnaire, and the general indicator^15^ ^,^ ^17^ ^-^ ^18^.

## Results

The majority of the specialists who participated in this study were aged between 50 and 59 years old (44.4%), were female 33 (91.7%) and resident in the South Region 15 (41.7%), Southeast 11 (30.6%), Center-West 5 (13.8%), Northeast 4 (11.1%) and North 1 (2.8%). The majority had graduated in the 1980s (52.8%), had undertaken *stricto sensu* training in public university institutions, were working as lecturers and in research, also in public universities, and had been working for 11 to 20 years (13: 36.0%), followed by 21 to 30 years (10; 27.8%). 

### First Delphi round 

In analyzing the level of alterations, in accordance with the three dimensions of the instrument for evaluating graduates, based on the specialists' responses to the questionnaire of the first round, it was ascertained that the majority of the items found in Dimension 1 - *Characterization of the graduate: identification and insertion in the labor market*, obtained a percentage below 70%, in the description of the items of this dimension, indicating a need for alterations. 

All the items present in Dimension 2: *Evaluation of the professional training process* (closed questions) and in Dimension 3: *Evaluation of the professional training process* (open questions) - in the first Delphi round also needed changes.

The results of the analysis of internal consistency of the items of the instrument used, in the first round of the Delphi Conference, are shown in Table 1. 

**Table 1:** Cronbach Alpha for the instrument of the first round of the Delphi Conference. São Paulo, State of São Paulo (SP), Brazil, 2014

Statistics of the scale		Mean of the indicator 8.42	Variance 34.993	Standard deviation 5.915	N. of items 3
Item	Statistical measurements item/total				
	The mean of the scale, if the item were excluded	Variants of the scale, if the item were excluded	Corrected correlation between item and total	Multiple correlation to the square	Alpha, if the item were excluded
Dimension 1	4.47	12.142	0.675	0.641	0.363
Dimension 2	5.64	18.580	0.382	0.185	0.777
Dimension 3	6.72	23.063	0.620	0.593	0.587
Alpha=0.694			Standardized items: alpha = 0.735		

According to Table 1, the Cronbach alpha value of 0.694 indicated that there was not consensus between the specialists and, consequently, calibration of the instrument in the first Delphi round did not take place. The mean of the scale of the indicator, if the item were excluded, would reduce to 4.47 in dimension 1, 5.64 in dimension 2, and 6.72 in dimension 3, showing that mere exclusion would not improve the quality of the instrument in this first round. The imperfection of the instrument could be perceived in the marked variability in the participants' responses, if the item were excluded. It also showed the weakness existing in the second dimension, which required care in its re-elaboration. In the evaluation of reliability, through the Pearson correlation coefficient, the best-adjusted relation was identified between the first and third dimension. However, the value found in the second dimension indicated a marked discrepancy between this block and the others, and this occurrence emphasized that the items of this dimension required reviewing for better calibration. Furthermore, the multiple correlation to the square showed that the second dimension expressed particular care in relation to possible changes in its content and form. 

As a result, various items of the questionnaire of the first round underwent changes in its three dimensions in order to respond to the statistical analyses and to the issues raised by the specialists, so that greater convergence could be obtained between the dimensions proposed in the questionnaire. In relation to the open questions of Dimension 3, the decision was made to re-do them and insert the Semantic Reference Scale, which has frequently been used for assessing the affective perception of people regarding objective and subjective situations, as a way of quantifying the affective meaning of the attitudes, opinions, perceptions, social image, personality, preferences and interests in relation to different contents^15^ ^,^ ^19^. In this study, this scale varied from 1 - inadequate through to 5 - adequate. 

### Second Delphi round 

The second round began in August 2013, and ended in September 2013, when the last questionnaires were returned by the specialists. The 36 specialists were sent the following: Explanatory Letter - a document with information on the statistical analyses undertaken and the decisions made by the researchers, and instructions referent to the second round; the Questionnaire of the Second Round of Application of the Delphi technique, and the expression of thanks for the participation and contributions made. A total of 34 questionnaires were received, with two people desisting, a fact which did not affect the validity and the quality of the study's results, as participants desisting is foreseen in the use of this technique, resulting in there being different numbers of participants in the rounds^19^.

The analyses of the specialists' responses to the questionnaire of the second round showed that, among the items found in Dimension 1 - *Characterization of the graduate: identification and insertion in the labor market*, only two items required changing. The other dimensions: *Evaluation of the professional training process* (closed and semistructured questions), in the second round of the Delphi Conference, did not require changes. The analysis of the internal consistency of the items of the instrument of the second Delphi round are described in Table 2. 

**Table 2:** Cronbach alpha for the Instrument of the Second Round of the Delphi Conference. São Paulo, SP, Brazil, 2014

Description of the item	Cronbach alpha, if the item were excluded
Identification	0.728
Do you, as a nurse, have a job in the area of nursing?	0.729
How long after graduating did you obtain your first job as a nurse?	0.751
Indicate how you succeeded in obtaining your current job as a nurse. Provide more than one alternative, if necessary	0.726
As a nurse, what is the total weekly hourly workload in your job? Please sum the hours worked per week if you have more than one job	0.729
What is your monthly net income from the work you exercise as a nurse?	0.724
Indicate the type of professional links that you have as a nurse. Indicate more than one alternative if necessary*	0.718
In relation to your current job as a nurse, indicate the function which you hold. You can indicate more than one alternative, if necessary	0.716
In what mode of healthcare do you exercise your job as a nurse? Indicate more than one alternative, if necessary	0.714
What is the nature of the institution where you exercise your job as a nurse? Indicate more than one alternative, if necessary	0.729
Have you undertaken, or are you undertaking, another course? Indicate more than one alternative, if necessary	0.730
Did the Undergraduate Course in Nursing prepare you to exercise the professional activities inherent to your area of work to be capable of:	0.720
Did the Undergraduate Course in Nursing instrumentalize you to assist the human being holistically, and thus to be capable of:	0.704
Did the Undergraduate Course in Nursing provide you with a basis, regarding ethical issues in the exercising of the profession, for you to be capable of:	0.713
Did the Undergraduate Course in Nursing prepare you technically and scientifically, to be capable of:	0.719
Did the Undergraduate Course in Nursing encourage the exercising of citizenship, for you to be capable of:	0.686
Did the Undergraduate Course in Nursing prepare you to undertake teaching activities and be capable of:	0.714
Did the Undergraduate Course in Nursing prepare you to work in a team and be capable of:	0.694
Did the Undergraduate Course in Nursing enable you to undertake management activities in healthcare and be capable of:	0.717
Consider your current job in Nursing and, if you have more than one, judge the main one, to respond to the following: How do you feel in relation to the Academic Training received on the Undergraduate Course in Nursing and the requirements experienced in the labor market?	0.729
How would you evaluate the Practical Experiences (placements) which you undertook on the Undergraduate Course in Nursing in relation to preparation for exercising the profession?	0.742
What is your perception in relation to the process of Evaluation adopted in the Undergraduate Course in Nursing which you undertook?	0.711
As a nurse, what is your assessment regarding your pay in Nursing?	0.718
How satisfied are you with the professional activities which you undertake in the area of Nursing?	0.726
How satisfied are you with being a nurse?	0.730
Considering your current professional perspectives, how would you evaluate the Undergraduate Course in Nursing which you undertook?	0.725
Alpha = 0.729	Standardized items: alpha = 0.750

*A nurse may be contracted on a variety of temporary or permanent bases, with significant differences in compensation and benefits. Translator's note.

Table 2 presented an alpha value = 0.750, indicating that consensus was reached in relation to that established previously. As a result, in this stage, it was ascertained that there was agreement among the specialists of the Delphi Conference, related to the internal consistency of the instrument for the evaluation of graduates from Undergraduate Courses in Nursing. The table also shows the Alpha values, were it decided to exclude the item. Note that only the item *How long after graduating did you obtain your first job as a nurse?* would improve the indicator, were it excluded from the questionnaire. This exclusion, however, would not improve the instrument significantly, given that the indicator would pass from 0.750 to 0.751. In this regard, the decision was made not to exclude it and rather to change this item's content in conformity with that suggested by the specialists and by the authors. These analyses, and some changes in the reconstruction of the second instrument, allow the construction and validation of the final data collection instrument for the evaluation of graduates of Undergraduate Courses in Nursing, termed the IAE-ENF, which can be found on the link below: https://drive.google.com/file/d/0B2ypGPriU5O1eUxRTGhBTmhMMFU/view

## Discussion

Considering that the Delphi panel must be made up of experts on the issue, which the literature widely states to be essential^19^ ^-^ ^20^, one can assert that this configuration was present in the quality of the composition of this study's Delphi Conference. It was identified that its participants possessed professional maturity, associated with extensive experience and the development of the ability to be socially critical and professionally self-critical, attested by their technical-scientific productions and by their employment links with well-known Brazilian public institutions. The Delphi technique was shown to be appropriate for this investigation's proposal and to be economically viable, allowed the participation of highly qualified professionals in an issue in which research is, as yet, incipient, and the performance of the technique online allowed speed in its conclusion and facilitated this participation. 

Most of the specialists live in the south and southeast of Brazil, where there is a greater concentration of programs, courses and academic investment^21^, but it is emphasized that there were representatives from all the Regions of Brazil, ensuring the inclusion of regional differences that also permeate the educational processes. 

Besides this, the adequate rate of response and/or return of the questionnaires in the second round - 94.44% - aggregated more power and strength to the validation process^22^. Emphasis is placed on the appropriate use of the Likert-type scale, in the second dimension, as the specialists gave opinions only in relation to the competencies and skills present in this dimension. Likewise, the Semantic Reference Scale, in the third dimension, was appropriate, as there was no need for changes in this dimension in the second round. Regarding the time taken to return the questionnaires, time-consuming in the first round, and faster in the second, this showed the commitment of the specialists who continued to participate in the Delphi Conference. 

One must emphasize that the IAE-ENF instrument is intended to reach all the graduates of a Higher Education Institution, regardless of how long it is since they graduated in Nursing, although it is considered that, if this was very long ago, it is possible that the graduate may have forgotten certain aspects of her professional training required to exercise the profession. Nevertheless, evaluating those who graduated long ago could bring the HEI data regarding academic training over the course of a professional trajectory, in the light of the challenges posed by the context of their work, considering that the theory offers what may be learned and understood by all, while the practice - the work - due to its uniqueness and complexity, provides richer and more productive learning in terms of meaning for the professional. In facing this reality, the graduate may compare the competences developed during the course with those required in exercising the profession, thus making her participation in an evaluative system - which allows the construction of an educational process with emancipatory and transformative characteristics - fundamental^23^. It is necessary for the educational practices in Nursing to be rethought, taking into account that the context and routine demonstrate that one must go beyond the teaching-learning process. It is necessary to transcend the academic concept of teaching which creates a professional ideology in Nursing which does not match the human and social reality experienced^24^. 

The HEI which chooses to use this instrument so as to include the graduate's opinion in its course evaluation process must define the time since qualification of the graduate whom it wishes to analyze, in accordance with its institutional objectives, which are found in the Pedagogical Course Project (PPC), the principal political and technical instrument for guiding the functioning of universities. The statistical analyses demonstrated the IAE-ENF to be valid and reliable because it is capable of measuring the variables which were raised through the use of the Delphi technique. According to each HEI's evaluation intentions, after the instrument has been applied in full, the items can be prioritized according to the institutional objectives, considering the specific characteristics of each HEI and the PPCs, making use of statistical resources for this purpose. 

## Conclusion

The instrument titled the IAF-ENF was constructed and validated, based on a wide review of the literature and on the DCN/ENF. Two Delphi rounds were held, with a Cronbach alpha coefficient value of 0.750, considered satisfactory in relation to that previously established. This proposal to further improve the evaluative system already existing in undergraduate Nursing training in Brazil, through the inclusion of the appreciation of the graduates in this system. The existence of a validated instrument must favor the adoption of the practice of the evaluation of the graduates in Brazilian HEIs and encourage further studies promoting its improvement. 

Limitations of the study: criteria validation was not undertaken, in which one seeks to assess the performance and behavior of the IAE-ENF with its users, in this case, the graduates. Another limitation may refer to the extent and breadth of the content of the IAE-ENF, in spite of these being intrinsic to the Curricular Guidelines for the area of Nursing. 
